# A Prospective Randomized Controlled Clinical Study to Investigate the Efficacy and Safety of Hypoxia-Inducible Factor-Prolyl Hydroxylase Inhibitors in Non-Dialysis Patients with Chronic Heart Failure and Renal Anemia Switched from Continuous Erythropoietin Receptor Activator Treatment

**DOI:** 10.3390/jcm13102764

**Published:** 2024-05-08

**Authors:** Akira Sezai, Masanori Abe, Takashi Maruyama, Makoto Taoka, Hisakuni Sekino, Masashi Tanaka

**Affiliations:** 1 Department of Cardiovascular Surgery, Nihon University School of Medicine, Tokyo 173-8610, Japan; taoka.makoto@nihon-u.ac.jp (M.T.); tanaka.masashi@nihon-u.ac.jp (M.T.); 2 Division of Nephrology, Hypertension and Endocrinology, Department of Internal Medicine, Nihon University School of Medicine, Tokyo 173-8610, Japan; abe.masanori@nihon-u.ac.jp (M.A.); maruyama.takashi@nihon-u.ac.jp (T.M.); 3 Sekino Hospital, Tokyo 171-0014, Japan; sekinoh@sekino-pre.sakura.ne.jp

**Keywords:** renal anemia, heart failure, hypoxia-inducible factor-prolyl hydroxylase inhibitor, erythropoiesis-stimulating agent, erythropoietin

## Abstract

**Background/Objectives**: Chronic kidney disease (CKD) and anemia are independent prognostic factors for heart failure. In recent years, hypoxia-inducible factor-prolyl hydroxylase (HIF-PH) inhibitors have become available for the treatment of renal anemia. This prospective randomized controlled study aimed to investigate the effects of switching from a continuous erythropoietin receptor activator (CERA) to one of four HIF-PH inhibitors in patients with chronic heart failure and renal anemia. **Methods**: Forty patients were randomized by the envelop method to receive treatment with roxadustat, daprodustat, vadadustat, or molidustat. The primary endpoint was the change in the hemoglobin (Hb) level. Secondary endpoints included changes in erythropoietin, changes in free T3, free T4, and thyroid-stimulating hormone (TSH), adverse effects, and drug dose increases and decreases. This study was preregistered in the University Hospital Medical Information Network Clinical Trials Registry (study ID: UMIN000041651). **Results**: We found no statistically significant difference between Hb levels with HIF-PH inhibitors and CERA, but at month 6, the Hb level was significantly higher with roxadustat than with vadadustat and daprodustat. Erythropoietin decreased significantly after switching to HIF-PH inhibitors. HIF-PH inhibitors had various significant effects on free T3, free T4, and TSH. No adverse events occurred. The doses of some drugs had to be increased or decreased. **Conclusions**: In patients with heart failure and renal anemia receiving CERA, Hb, NT-ProBNP, and renal function were similar after switching from CERA to HIF-PH inhibitors. The individual HIF-PH inhibitors appear to have different effects on anemia and thyroid function. However, because this was a single-center study with a limited sample size, the efficacy and potential limitations of HIF-PH inhibitors need to be further clarified.

## 1. Introduction

Chronic kidney disease (CKD) and anemia are prognostic factors for poor outcomes of heart failure [1], and this tri-partite relationship may enter a vicious cycle referred to as cardiorenal anemia syndrome [1]. Erythropoiesis-stimulating agents (ESAs) are used to treat renal anemia and are reported to improve the New York Heart Association functional classification, decrease hospital readmission rates for heart failure, and increase exercise tolerance [2,3,4]. However, although the Red-HF (Reduction of Events by Darbepoetin Alfa in Heart Failure) study, a large-scale clinical study of ESAs in patients with cardiorenal anemia syndrome, showed improvement in hemoglobin (Hb) in the ESA group, the primary endpoint (all-cause death or re-admission because of worsening of heart failure) was not significantly different from the placebo group. On the other hand, the incidence of thromboembolism was reported to be higher in the ESA group [5]. Thus, the efficacy of ESAs in heart failure is not established. Previously, we reported the results of a clinical study of continuous erythropoietin receptor activators (CERAs) in patients with cardiorenal anemia syndrome in which CERAs not only improved anemia but also reduced renal impairment, cardiac stress, and oxidative stress [6].

Recently, hypoxia-inducible factor-prolyl hydroxylase (HIF-PH) inhibitors have become available as a treatment for renal anemia. They are administered orally and increase the production of endogenous erythropoietin. Currently, five HIF-PH inhibitors (vadadustat, daprodustat, roxadustat, enarodustat, molidustat) are available for clinical use in both dialysis patients and non-dialysis patients with CKD in Japan. Comparative studies of daprodustat versus ESAs (ASCEND-D [Anemia Studies in Chronic Kidney Disease: Erythropoiesis Via a Novel Prolyl Hydroxylase Inhibitor Daprodustat-Dialysis] in dialysis patients [7] and ASCEND-ND [Anemia Studies in Chronic Kidney Disease: Erythropoiesis Via a Novel Prolyl Hydroxylase Inhibitor Daprodustat-Non-Dialysis] in non-dialysis patients with CKD [8]) showed inferior Hb changes and cardiovascular outcomes with ESAs.

We performed a prospective, randomized, controlled study to investigate the effects of switching patients with chronic heart failure and renal anemia being treated with CERAs to four different types of HIF-PH inhibitors. The primary endpoint was a change in Hb levels, and the secondary endpoints included markers of heart function; renal, iron, and thyroid parameters; adverse events; and dose changes. To our knowledge, this was the first head-to-head comparative study of HIF-PH inhibitors.

## 2. Materials and Methods

### 2.1. Participants

This study enrolled male and female patients aged 69 to 90 years who had chronic heart failure and renal anemia, who had been receiving a CERA for renal anemia for at least 12 months, and who were being treated at Sekino Hospital, a logistical support hospital of the Nihon University Itabashi Hospital, Tokyo, Japan. Patients were observed for 6 months over the period from October 2020 to September 2021. This study was single-blind, i.e., the investigators were blind to the treatment group.

Chronic heart failure was defined as a history of heart failure in patients currently on guideline-directed medical therapy for heart failure, and renal anemia was defined as an Hb level of 11 g/dL or less (corresponding to mild anemia); CKD stage 3b or lower; negative fecal occult blood test results; anemia that was not iron deficiency, pernicious, or hemolytic anemia; no vitamin B_12_ or folic acid deficiency; or a diagnosis of iron deficiency anemia that did not improve despite administration of an iron preparation.

Exclusion criteria were as follows: (1) hemodialysis, (2) hepatic dysfunction, (3) pregnancy, and (4) patients who were judged by the attending physician to be unsuitable for this study.

**Figure 1:**
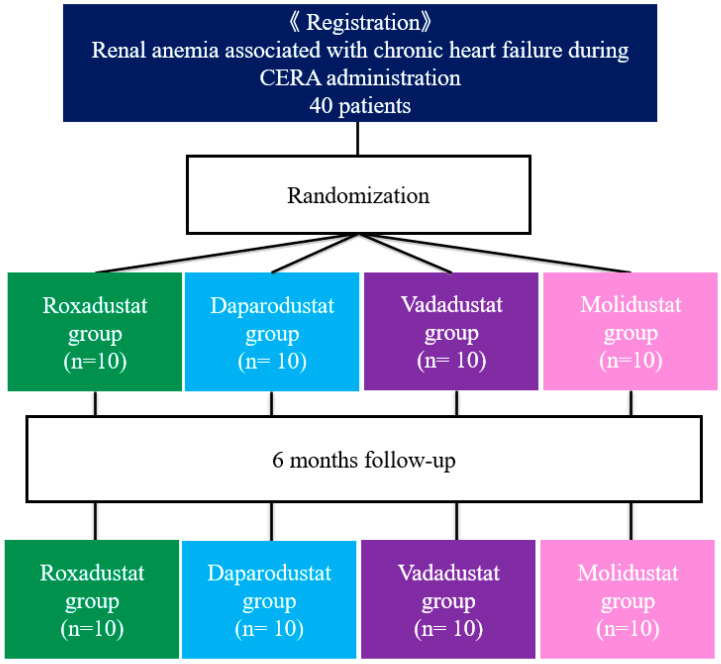
Study design and patient flow through this study.

Patients were randomized by the envelop method to receive treatment with roxadustat (Astellas Pharma Inc., Tokyo, Japan), daprodustat (Kyowa Kirin Co., Ltd., Tokyo, Japan), vadadustat (Mitsubishi Tanabe Parma Co., Osaka, Japan), or molidustat (Bayer Yakuhin, Ltd., Osaka, Japan) for 6 months under the management of an outpatient primary care physician (Figure 1). After assignment to the intervention, the primary care physician was blinded to the type of drug. After a two-week wash-out period after discontinuation of the CERA, the initial dose of HIF-PH inhibitor recommended by the drug manufacturer (roxadustat: 70 mg/day, 3 times weekly; daprodustat: 4 mg/day; vadadustat: 300 mg/day; molidustat: 25 mg/day) was administered. Thereafter, the dose was adjusted to maintain the Hb level in the range of 11 to about 13 g/dL; there is no target Hb range for HIF-PH inhibitors, so in this study, we used the Hb range of 11–13 g/dL recommended for ESA treatment for renal anemia in the Japanese Evidence-based Clinical Practice Guideline for Chronic Kidney Disease. Each HIF-PH inhibitor is indicated for the conservative treatment of renal anemia in Japan and is covered by health insurance. Informed consent was obtained from patients after informing them that the HIF-PH inhibitor was for the treatment of renal anemia.

This study was approved by the Sekino Hospital institutional review board and was preregistered in the University Hospital Medical Information Network (study ID: UMIN000041651). Written informed consent was obtained from all participants.

### 2.2. Endpoints

The primary endpoint was the change in Hb level after 1, 3, and 6 months of treatment with an HIF-PH inhibitor. The secondary endpoints were as follows: (1) change at 3 and 6 months in N-terminal prohormone of brain natriuretic peptide (NT-ProBNP); (2) change at 3 and 6 months in serum creatinine (sCr) and estimated glomerular filtration rate (eGFR); (3) change at 3 and 6 months in erythropoietin; (4) change at 3 and 6 months in transferrin saturation (TSAT) and ferritin; (5) change at 3 and 6 months in free T3, free T4, and thyroid-stimulating hormone (TSH); (6) adverse effects; and (7) drug dose increases or decreases. Changes were calculated by comparing data obtained during CERA treatment with those obtained after switching to an HIF-PH inhibitor.

Adverse effects of HIF-PH inhibitors include hypertension, tachycardia, bradycardia, renal dysfunction (defined as an increase in serum creatinine levels of about 50%), hepatic dysfunction (defined as an increase in aspartate transaminase or alanine transaminase levels of about 50%, skin reactions, and allergic reactions. Management of adverse events, such as discontinuation of the test drug, was determined by the attending physician.

### 2.3. Statistical Analysis

Data are presented as median and interquartile range (25th and 75th percentile). Values from before and after the switch from CERA were compared by the Friedman rank test or Kruskal–Wallis test and HIF-PH inhibitors were compared with the Wilcoxon signed rank test. A *p*-value of less than 0.05 indicated statistical significance. All analyses were performed with SPSS software Ver.28.0 (IBM SPSS Statistics, Chicago, IL, USA). Data were aggregated by Sekino Laboratory staff who were not involved in this study, and statistical analyses were supported by Data Seed Inc. (Tokyo, Japan), a company not involved in performing this study.

## 3. Results

### 3.1. Patients

Forty patients were enrolled, randomly assigned to receive the intended treatment, and analyzed for the primary outcome. Patient characteristics are shown in Table 1, and data obtained during CERA treatment are shown in Table 2. This study continued for 6 months, and no adverse events or worsening of heart failure or renal function, thromboembolism, malignancy, or retinal hemorrhage occurred.

During the study period, the following changes were made to the dose of HIF-PH inhibitors: roxadustat, dose increase in zero patients and dose decrease in four patients (40%); daprodustat, dose increase in six patients (60%) and dose decrease in zero patients; vadadustat, dose increase in six patients (60%) and dose of decrease in one patient (10%); and molidustat, dose increase in three patients (30%) and dose decrease in three patients (30%).

### 3.2. Primary Endpoint

**Figure 2:**
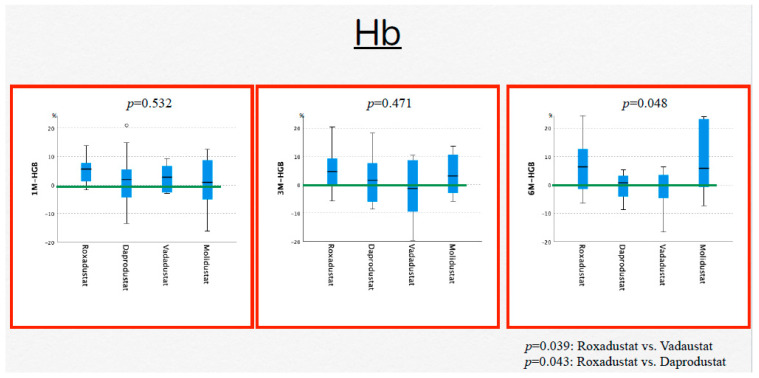
Changes in the levels of hemoglobin: 1M-HGB, level of hemoglobin at 1 month; 3M-HGB, level of hemoglobin at 3 months; and 6M-HGB, level of hemoglobin at 6 months.

Hb levels showed no statistically significant change from the value during CERA treatment to that during HIF-PH inhibitor treatment as follows: roxadustat, *p* = 0.128; daprodustat, *p* = 0.751; vadadustat, *p* = 0.431; and molidustat, *p* = 0.238. In the comparison of Hb changes between the four HIF-PH inhibitors, there was no statistically significant difference between the drugs at month 1 (*p* = 0.532) or 3 (*p* = 0.471); however, at month 6, a statistically significant difference was found (*p* = 0.048), and Hb increased significantly more with roxadustat than with vadadustat (*p* = 0.039) and daprodustat (*p* = 0.043) (Figure 2).

### 3.3. Secondary Endpoints

The changes in secondary endpoints after 3 and 6 months of treatment with an HIF-PH inhibitor are shown in Table 3 and discussed in detail below.

#### 3.3.1. NT-ProBNP

After switching from CERA, NT-ProBNP showed no statistically significant change with any of the HIF-PH inhibitors (roxadustat, 0.301; daprodustat, *p* = 0.497; vadadustat, *p* = 0.122; and molidustat, *p* = 0.497). After 3 and 6 months of treatment with an HIF-PH inhibitor, NT-ProBNP was also not significantly different between the drugs (3 months, *p* = 0.464; 6 months, *p* = 0.638)

#### 3.3.2. sCr and eGFR

After switching from CERA, sCR showed no statistically significant change with any of the HIF-PH inhibitors (roxadustat, 0.258; daprodustat, *p* = 0.292; vadadustat, *p* = 0.401; and molidustat, *p* = 0.421). After 3 and 6 months of treatment with an HIF-PH inhibitor, sCr was also not significantly different between the drugs (3 months, *p* = 0.268; 6 months, *p* = 0.322).

The same results were found for eGFR before and after the switch to HIF-PH inhibitors (roxadustat, *p* = 0.258; daprodustat, *p* = 0.239; vadadustat, *p* = 0.339; and molidustat, *p* = 0.421) and after 3 and 6 months (3 months, *p* = 0.474; 6 months, *p* = 0.954).

#### 3.3.3. Erythropoietin

With all HIF-PH inhibitors, erythropoietin significantly decreased during treatment compared with before the switch from CERA (roxadustat, pre vs. 3 months, *p* = 0.002, and pre vs. 6 months, *p* < 0.001; daprodustat, pre vs. 3 months, *p* = 0.004, and pre vs. 6 months, *p* = 0.014; vadadustat, pre vs. 3 months, *p* = 0.01, and pre vs. 6 months, *p* = 0.034; and molidustat, pre vs. 3 months, *p* = 0.007, and pre vs. 6 months, *p* < 0.001). After 3 and 6 months of treatment with an HIF-PH inhibitor, erythropoietin was not significantly different between the drugs (3 months, *p* = 0.208; 6 months, *p* = 0.956).

#### 3.3.4. TSAT and Ferritin

There was no statistically significant change in TSAT with any of the HIF-PH inhibitors after switching from CERA (roxadustat, *p* = 0.729; daprodustat, *p* = 0.670; vadadustat, *p* = 0.202; and molidustat, *p* = 0.407). After 3 and 6 months of treatment with HIF-PH inhibitors, TSAT was also not significantly different between the drugs (3 months, *p* = 0.965; 6 months, *p* = 0.930).

For ferritin, no statistically significant change was found with any of the HIF-PH inhibitors after switching from CERA (roxadustat, *p* = 0.052; daprodustat, *p* = 0.670; vadadustat, *p* = 0.717; and molidustat, *p* = 0.836). After 3 and 6 months of treatment with HIF-PH inhibitors, ferritin was not significantly different between the drugs (3 months, *p* = 0.228; 6 months, *p* = 0.533).

#### 3.3.5. Free T3, Free T4, and TSH

There was a significant decrease in free T3 with roxadustat at month 3 after switching from CERA (*p* < 0.001), but the difference was no longer significant at month 6 (*p* = 0.057). For the other HIF-PH inhibitors, there was no statistically significant change in free T3 after switching from CERA (daprodustat, *p* = 0.061; vadadustat, *p* = 0.094; and molidustat, *p* = 0.209).

Free T4 showed no statistically significant change with any of the HIF-PH inhibitors after switching from CERA (roxadustat, *p* = 0.135; daprodustat, *p* = 0.301; vadadustat, *p* = 0.908; and molidustat, *p* = 0.741).

TSH was significantly lower with roxadustat at month 3 (*p* < 0.001), but the difference was no longer significant at month 6 (*p* = 0.118) (Figure 2). For the other HIF-PH inhibitors, there was no statistically significant change in TSH after switching from CERA (daprodustat, *p* = 0.122; vadadustat, *p* = 0.216; and molidustat, *p* = 0.819).

**Figure 3:**
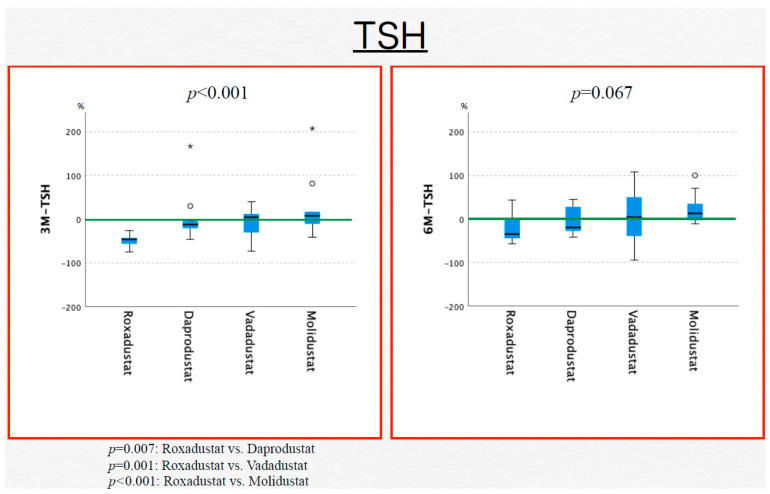
Changes in the levels of thyroid-stimulating hormone after 3 and 6 months of treatment with a hypoxia-inducible factor-prolyl hydroxylase inhibitor: 3M-TSH, thyroid-stimulating hormone level at 3 months, and 6M-TSH, thyroid-stimulating hormone level at 6 months.

At month 3 after initiating HIF-PH inhibitor treatment, free T3, free T4, and TSH were significantly decreased with roxadustat compared with the other HIF-PH inhibitors. At month 3, free T3 was significantly decreased with roxadustat compared with daprodustat (*p* = 0.009) and molidustat (*p* < 0.001) and was significantly decreased with vadadustat compared with molidustat (*p* = 0.001), and at month 6, it was significantly increased with molidustat compared with roxadustat (*p* = 0.002) and with vadadustat compared with molidustat (*p* = 0.001). At month 3, free T4 was significantly lower with roxadustat than with daprodustat (*p* = 0.009) and molidustat (*p* = 0.022), but there was no significant difference between the drugs at month 6. At month 3, TSH was significantly lower with roxadustat than with the other three drugs (daprodustat, *p* = 0.007; vadadustat, *p* = 0.001; and molidustat, *p* < 0.001), but there was no significant difference between the drugs at month 6 (*p* = 0.067) (Figure 3).

## 4. Discussion

In this study, patients with chronic heart failure and renal anemia were switched from a CERA to an HIF-PH inhibitor. No adverse events were observed with any of the HIF-PH inhibitors, and all participants completed treatment safely. Moreover, HIF-PH inhibitors showed equal efficacy to CERA in increasing Hb levels. Although Hb increased significantly more with roxadustat than with daprodustat and vadadustat, the dose had to be reduced in 40% of the patients. The finding that the HIF-PH inhibitor dose had to be increased or decreased in some patients indicates that Hb needs to be monitored in all patients on HIF-PH inhibitors and drug doses need to be adjusted so that the level is maintained at 11 to about 13 g/dL to prevent the onset of thromboembolism.

Currently, five different HIF-PH inhibitors are available in Japan, including the four drugs evaluated in this study and enarodustat (Torii Pharm Co., Ltd., Tokyo, Japan). To date, no study has directly compared the HIF-PH inhibitors, highlighting the importance of this work. However, because the study design included a switch from CERA treatment, a future study is needed to compare the HIF-PH inhibitors in treatment-naïve patients with renal anemia and thus further characterize the individual drugs.

Few studies have evaluated HIF-PH inhibitors in patients with chronic heart failure and renal anemia. The first case report was published by Imamura et al. in 2021 and described a patient with renal anemia complications who received daprodustat for 6 months for the management of heart failure with preserved ejection fraction; Hb was reported to improve from 8.8 to 10.4 g/dL and B-type natriuretic peptide from 276 to 122 pg/mL [9]. The same group described another patient with renal anemia complications who received daprodustat for the management of heart failure with mid-range ejection fraction for 7 months. Improvements were seen in Hb (from 7.4 to 11.8 g/dL), eGFR (from 24 to 35 mL/min/1.73 m^2^), left ventricular ejection fraction (from 46% to 54%), 6-minute walk distance, and the Barthel index [10]. A prospective study evaluated 13 patients with heart failure and renal anemia complications and included patients who switched from ESA; 3 patients discontinued treatment because of headache and hot flashes, but 10 patients were followed for 3 months [11]. Hb improved significantly, and hepcidin-25 appeared to decrease. The authors concluded that HIF-PH inhibitors appear to be safe and effective for the treatment of renal anemia in patients with chronic heart failure [11]. Currently, Iso et al. are conducting a pilot, multi-center, open-label, randomized controlled study to investigate the effects of daprodustat in patients with chronic heart failure and renal anemia complications [12].

Although the efficacy of HIF-PH inhibitors in patients with chronic heart failure and renal anemia has not yet been clearly demonstrated, improvements in Hb levels may be an effective approach to evaluate the effects of these drugs in cardiorenal anemia syndrome. Regarding ESA, the Red-HF study demonstrated an improvement in Hb in the ESA group, but the primary endpoint (all-cause death and re-admission because of worsening of heart failure) was not significantly different from the placebo group [5]. Furthermore, 72% of patients in the ESA group had an Hb level higher than 13 g/dl three times, implying that Hb levels were not carefully monitored in the study. Nevertheless, the number of readmissions due to heart failure was lower in the ESA group (albeit not significantly lower [*p* = 0.06]). The authors suggested that an improvement in moderate or severe anemia by ESA may prevent the worsening of heart failure. Similar effects are anticipated for HIF-PH inhibitors.

Concerning thyroid hormones, the present study found that free T3, free T4, and TSH were significantly lower with roxadustat than with the other HIF-PH inhibitors. However, none of the participants had thyroid problems, and none of them required treatment for thyroid issues. With regard to the effects of roxadustat on the thyroid, Haraguchi et al. performed a retrospective evaluation of thyroid function (TSH, free T4) in patients who received daprodustat or roxadustat. Those receiving daprodustat did not show any changes from before to after treatment, and no patient demonstrated hypothyroidism symptoms in the roxadustat group; however, the authors reported a significant decrease in TSH and free T4 after treatment with this roxadustat [13]. Tokuyama et al. reported a rapid decrease in TSH and free T3 in hemodialysis patients with hypothyroidism receiving treatment with levothyroxine after they were switched from daprodustat to roxadustat and found that the hypothyroidism disappeared after roxadustat was discontinued. The authors stated that roxadustat may suppress TSH release because it has structural similarities with T3 and is a selective activating ligand for thyroid hormone receptor-b [14].

Concerning iron metabolism, a comparative study of ESA and an HIF-PH inhibitor found that hepcidin and ferritin decreased significantly more with the HIF-PH inhibitor than with the ESA, but the effects of both drugs on TSAT were comparable. The study also found that the HIP-PH inhibitor improved iron utilization [15]. The present study did not find a difference in iron metabolism compared with CERA and found no difference among the HIF-PH inhibitors. TSAT and ferritin data showed variations, which need to be clarified in the future.

In the present study, the concentration of erythropoietin decreased significantly after switching to any HIF-PH inhibitor from CERA. The concentration of erythropoietin was measured in all patients 1 month after treatment with CERA. Considering the half-life of erythropoietin, the concentration of erythropoietin is expected to be high immediately after discontinuation of CERA and to decrease at 1 month after discontinuation. However, after administration of HIF-PH inhibitors, the level was consistently significantly lower than during CERA administration. Rapid elevation of the erythropoietin concentration may result in a sudden increase in Hb, which may increase the risk of thromboembolism. Accordingly, compared with CERA, HIF-PH inhibitors are hypothesized to maintain a physiological concentration of erythropoietin and thereby reduce the risk of thromboembolism. This hypothesis needs to be further studied as the number of patients treated with HIF-PH inhibitors increases.

## 5. Conclusions

In patients with heart failure and renal anemia receiving CERA, Hb, NT-ProBNP, and renal function were similar after switching from CERA to an HIF-PH inhibitor. Each HIF-PH inhibitor may exert different effects on Hb and thyroid function. However, this study had a small sample size and was performed at a single center. Larger numbers of patients need to be studied to clarify the efficacy and potential limitations of HIF-PH inhibitors.

## 6. Limitations

This study has several limitations. First, it had a small sample size and used a single-center design. Second, it did not include a placebo arm. Although the efficacy of each drug was previously demonstrated in large-scale clinical studies, a placebo-controlled study that includes all HIF-PH inhibitors is needed to demonstrate the efficacy of these drugs. Last, because this was a study in the field of heart failure research, we should have performed stratified analyses by LVEF subtypes (HFrEF, HFmrEF, HFpEF); however, we were unable to perform such analyses because of the limited number of patients. Additional studies are needed to further investigate the effects of HIF-PH inhibitor treatment and clarify the generalizability of the findings.

## Figures and Tables

**Table 1:** Background data.

	Roxadustat	Daparodustat	Vadadustat	Molidustat
Number	10	10	10	10
Age (years)	81.0 (78.8–88.3)	83.0 (76.5–88.0)	80.0 (74.3–84.8)	83.5 (78.3–88.3)
Gender (male–female)	7:3	6:4	7:3	7:3
Basic disease				
Ischemic heart disease	4 (40%)	3 (30%)	5 (50%)	3 (30%)
Valvular disease	4 (40%)	3 (30%)	3 (30%)	3 (30%)
Hypertensive heart disease	2 (20%)	2 (20%)	2 (20%)	2 (20%)
Cardiomyopathy	0	2 (20%)	0	2 (20%)
Classification of heart failure				
HFrEF	3 (30%)	2 (20%)	3 (30%)	1 (10%)
HFmrEF	2 (20%)	5 (50%)	2 (20%)	5 (50%)
HFpEF	5 (50%)	3 (30%)	5 (50%)	4 (40%)
CERA dose (μg)	75.0 (68.8–81.3)	75.0 (50.0–103.1)	75.0 (62.5–100.0)	75.0 (68.8–100.0)

CERA, continuous erythropoietin receptor activator; HFmrEF, heart failure with mid-range ejection fraction; HFpEF, heart failure with preserved ejection fraction; HFrEF, heart failure with reduced ejection fraction.

**Table 2:** Data before administration of a hypoxia-inducible factor-prolyl hydroxylase inhibitor. Data are shown as the median (interquartile range).

	Values with Each Hypoxia-Inducible Factor-Prolyl Hydroxylase Inhibitor			
	Roxadustat (n = 10)	Daprodustat (n = 10)	Vadadustat (n = 10)	Molidustat (n = 10)
Hemoglobin, g/dL	12.7 (11.4–13.0)	12.3 (11.9–13.1)	11.9 (11.4–12.4)	12.3 (11.6–13.2)
NT-ProBNP, pg/mL	1292.3 (461.4–3166.1)	1252.4 (430.9–1897.7)	1206.1 (866.6–3216.9)	886.3 (388.1–2738.1)
Creatine, mg/dL	1.48 (1.20–1.86)	1.61 (1.21–1.97)	1.78 (1.45–1.92)	1.47 (1.16–1.83)
eGFR, mL/min/1.73 m^2^	32.8 (22.1–36.6)	32.3 (21.9–34.3)	27.2 (20.4–34.8)	33.0 (26.2–40.9)
Erythropoietin, mIU/mL	64.5 (35.5–109.0)	46.7 (23.3–122.3)	42.1 (26.2–67.1)	48.0 (38.7–118.0)
TSAT, %	31.9 (16.6–47.0)	37.3 (23.9–49.9)	27.9 (21.7–47.3)	27.5 (20.7–41.1)
Ferritin, ng/mL	192.0 (80.3–384.0)	131.0 (50.0–275.5)	163.0 (118.5–297.5)	161.5 (110.5–253.0)
Free T3	1.2 (1.1–1.2)	1.3 (1.2–1.4)	1.3 (1.1–1.4)	1.2(1.0–1.3)
Free T4	2.5 (2.3–3.0)	2.6 (2.2–3.0)	2.3 (2.0–2.5)	2.6 (2.4–3.0)
TSH	2.7 (1.8–7.3)	2.8 (1.8–7.0)	2.5 (2.3–3.7)	3.9 (3.4–6.8)

eGFR, estimated glomerular filtration rate; NT-ProBNP, N-terminal prohormone of brain natriuretic peptide; TSAT, transferrin saturation; TSH, thyroid-stimulating hormone.

**Table 3:** Percentage change in secondary endpoints after starting treatment with a hypoxia-inducible factor-prolyl hydroxylase inhibitor. Data are shown as the median (interquartile range).

	Change after 3 and 6 Months’ Treatment with Each Hypoxia-Inducible Factor-Prolyl hydroxylase Inhibitor							
	Roxadustat		Daprodustat		Vadadustat		Molidustat	
	3 Months	6 Months	3 Months	6 Months	3 Months	6 Months	3 Months	6 Months
NT-ProBNP, %	−30.7 (−49.1 to 5.7)	−9.6 (−44.5 to 48.4)	5.5 (−40.1 to 16.9)	6.6 (−20.3 to 49.6)	−14.2 (−22.3 to 34.1)	6.4 (−15.9 to 68.3)	−21.9 (−37.9 to 18.9)	−12.3 (−46.4 to 35.0)
sCr, %	11.6 (−0.9 to 28.2)	9.0 (−3.3 to 20.6)	0.6 (−8.0 to 11.2)	10.6 (−6.7 to 20.6)	9.7 (1.9–13.7)	3.3 (−3.3 to 17.4)	6.1 (−4.1 to 13.5)	10.8 (−14.1 to 18.5)
eGFR, %	−11.0 (−23.7 to 0.9)	−9.2 (−18.7 to 3.5)	−0.6 (−10.8 to 9.6)	−10.6 (−18.8 to 7.9)	−9.7 (−13.4 to 2.0)	−3.6 (−15.8 to 3.7)	−6.3 (−12.6 to 4.9)	−10.8 (−17.1 to 18.0)
EPO, %	−59.2 (−74.2 to −39.0)	−70.7 (−86.3 to −41.0)	−67.4 (−80.8 to −40.8)	−65.4 (−81.8 to −58.2)	−50.7 (−68.1 to −20.3)	−58.4 (−80.3 to −1.6)	−51.6 (−71.3 to −1.0)	−62.0 (−84.3 to −3.3)
TSAT, %	−7.6 (−31.9 to 30.5)	−5.8 (−40.9 to 47.0)	−10.1 (−16.1 to 32.5)	−3.6 (−33.1 to 27.19	−15.2 (−23.0 to 102.2)	−21.3 (25.0 to 13.4)	25.8 (−60.5 to 47.1)	9.2 (−38.8 to 57.9)
Ferritin, %	−27.4 (−35.4 to 5.2)	−8.8 (−38.0 to 30.6)	17.9 (−27.7 to 39.4)	−6.4 (−31.9 to 89.2)	−2.5 (−18.7 to5 0.7)	−15.5 (−37.0 to 53.4)	5.7 (−26.4 to 26.5)	24.3 (−31.7 to 92.4)
Free T3, %	−23.8 (−29.4 to −13.9)	−18.8 (−30.8 to 0.9)	−8.8 (−13.7 to −0.7)	−4.0 (−15.6 to 0)	0 (−4.9 to 0.9)	−0.1 (−6.5 to 0)	3.5 (−0.9 to 22.6)	5.8 (−5.3 to 18.5)
Free T4, %	−14.0 (−20.1 to −5.8)	−10.9 (−26.8 to 11.3)	5.3 (−3.2 to 17.7)	6.0 (−1.3 to19.0)	−0.2 (−4.3 to 10.1)	0.2 (−4.4 to 2.5)	5.2 (−2.1 to 18.5)	2.8 (−14.4 to 8.7)

NT-ProBNP: N-terminal prohormone of brain natriuretic peptide, sCr: serum creatinine, eGFR: estimated glomerular filtration rate, EPO: erythropoietin, TSAT: transferrin saturation.

## Data Availability

The data that support the findings of this study are available on request from the corresponding author, [A.S]. The data are not publicly available due to [restrictions e.g., their containing information that could compromise the privacy of research participants].
